# High antioxidant and DNA protection activities of *N*-acetylglucosamine (GlcNAc) and chitobiose produced by exolytic chitinase from *Bacillus cereus* EW5

**DOI:** 10.1186/2193-1801-3-354

**Published:** 2014-07-11

**Authors:** Mohammed Shariful Azam, Eun Jung Kim, Han-Soeb Yang, Joong Kyun Kim

**Affiliations:** Department of Biotechnology, Pukyong National University, 45 Yongso-Ro, Nam-Gu, Busan, 608-737 Korea; Department of Oceanography, Environmental and Marine Sciences and Technology, Pukyong National University, 45 Yongso-Ro, Nam-Gu, Busan, 608-737 Korea

**Keywords:** *Bacillus cereus*, GlcNAc, Antioxidant, DNA protection, Exochitinase

## Abstract

Chitin-degrading bacterial strains were screened and tested for their ability to degrade shrimp-shell waste (SSW). Among the potential strains, *B. cereus* EW5 exhibited the highest chitin-degrading ability compared with other strains and produced 24 mg of reducing sugar per gram of dry SSW after 4 days of incubation. A TLC analysis of SSW biodegradation revealed that the chitosaccharides produced in the culture supernatant were mainly *N*-acetylglucosamine (GlcNAc) and chitobiose due to the isolate’s exolytic chitinase activity. The culture supernatant exhibited a high degree of antioxidant activity, as indicated by 83% DPPH, 99.6% ABTS, 51% hydroxyl radical scavenging activity and 0.34 reducing power. The formation of GlcNAc and chitobiose during biodegradation of SSW is considered to be the major contributor to the antioxidant activity. The EW5 culture supernatant also displayed inhibition of DNA damage, enhancing the reutilization value of SSW. This report presents the first description of fermented production of GlcNAc and DNA protective activity of culture supernatant from SSW by *B. cereus*.

## Background

Shrimp processing waste is one of the main byproducts of fishery industries in Asia. The continent plays a leading role in shrimp farming, accounting for approximately 80% of the world shrimp production (Fuchs et al. 1999). The increasing demand for farmed shrimp production and processing in the world market has led to increased waste generation. Among the Asian nations, China, Bangladesh and India produce approximately 150, 114 and 100 thousand tons of shrimp waste per year, respectively (Khan and Hossain 1996; Liu and Ye 2007; Suresh and Kumar 2012). Major portions of these wastes remain unutilized and are disposed of in landfills or dumped into the sea. These wastes create bad smells and greatly pollute the environment (Nargis et al. 2006), producing a significant adverse effect on ecosystems (Suresh 2012). Therefore, potential utilization techniques for shrimp waste should be established not only to solve the environmental pollution problem but also to obtain high-value biomolecules.

Among natural resources, shrimp shells have the highest content of chitin, the most abundant biopolymer in nature after cellulose (Chen et al. 2010). However, the biopolymer is still underutilized because of its crystalline nature and insolubility in aqueous media. Bellaaj et al. ( 2011) reported that the protein, fat, ash and chitin contents of the dry weight of shrimp shells were 24.9 ± 0.7%, 6.2 ± 0.3%, 46.1 ± 0.6% and 25.2 ± 1.9%, respectively. Recent studies have focused on the conversion of chitin into chitooligosaccharides because of its water-solubility and diverse functional properties, such as antitumor activity (Liang et al. 2007), antimicrobial activity (Arancibia et al. 2014; Benhabiles et al. 2012) and antioxidant activity (Annamalai et al. 2011; Arancibia et al. 2014). *N*-acetylglucosamine (GlcNAc), monomer of chitin, has great potential in the treatment of several diseases, including osteoarthritis (Talent and Gracy 1996), joint injury (Tamai et al. 2003), gastritis and inflammatory bowel disease (Chen et al. 2010).

Traditionally, chitin and chitosaccharides are produced industrially by chemical methods. However, the traditional chemical process results in the formation of undesired byproducts and creates large quantities of toxic waste that require further treatment to avoid environment pollution (Sini et al. 2007). The oligosaccharides produced by acidic hydrolysis can be toxic due to chemical changes during treatment. The GlcNAc produced by chemical methods is also not considered a natural material due to its chemical modification (Sashiwa et al. 2001). Poor repeatability and difficulty in controlling reaction conditions, the cost of the chemicals, low yield of oligosaccharides and the high cost of separation are other drawbacks of this approach (Wang et al. 2012). To overcome the problem of chemical treatment, biological processes, such as bacterial fermentation (Bellaaj et al. 2011) or enzymatic treatment (Manni et al. 2010), have been suggested as an environmentally friendly method. However, microbial fermentation is advantageous over enzyme hydrolysis, as this process omits the procedure for purifying enzymes and reduces the cost (Wang et al. 2012).

Currently, there is an increasing interest in antioxidants from natural, rather than synthetic, sources (Abdalla and Roozen 1999), as the antioxidants play important roles in protecting key cellular components, such as lipids, proteins and DNA, by neutralizing free radical-induced damage in humans (Shenoy and Shirwaikar 2002). Seymour et al. ( 1996) reported that shrimp waste contains natural antioxidants, primarily phenolic compounds. To date, SSW has been used mostly for the production of chitin (Bellaaj et al. 2012a; Zhang et al. 2012), chitosanases (Wang et al. 2009b) and antifungal chitinases (Halder et al. 2013) via bacterial fermentation. Several studies have reported on the bacterial fermentation production of antioxidants from SSW (Bellaaj et al. 2012a; Wang et al. 2009a). However, most of these studies reported the antioxidant activity of the SSW hydrolysates might be due to the chitooligosaccharides and peptides produced during fermentation. Recently, Halder et al. ( 2013) reported the potential antioxidant activity of GlcNAc and chitobiose produced by biodegradation of SSW using *Aeromonas hydrophila*. Nevertheless, there is scant information available on the biodegradation of SSW into GlcNAc and chitobiose by bacterial fermentation and the antioxidant activity of these products. For this reason, the present study attempted to screen chitin-degrading bacteria for the recovery of the valuable natural antioxidants GlcNAc and chitobiose from SSW. In this study, we also investigated the protective effect of biodegraded SSW against DNA damage to increase the reutilization value of SSW.

## Results and discussion

### Screening of chitin-degrading strains

After 8 days of incubation, seven and six different types of colonies were isolated from the tidal mud and shrimp pond soil, respectively. Two isolates from the tidal mud, designated TM1 and TM2, and three isolates from pond soil, designated SPS1, SPS2 and SPS3, displayed positive growth on SSP agar after 2 days of incubation. Among our laboratory-stored eight strains tested for their chitin-degrading ability on SSP agar, only two strains, *Bacillus cereus* EW5 (GenBank accession no. DQ923487) and *B. subtilis* KA1 (GenBank accession no. DQ219358) displayed positive growth.

### Chitinase activity

For the seven strains displaying positive growth on SSP agar, the chitin-degrading ability was evaluated using Lugol’s solution. After 4 days of incubation, all strains, except EW5, produced a clear zone on the SSP agar, with the largest diameter of 4.9 cm by KA1 followed by 4.5 cm and 4.2 cm from TM2 and SPS3, respectively.

### Identification and characterization of useful chitin-degrading strains

According to the results of 16S rDNA nucleotide sequencing, TM2 and SPS3 strains were identified as *Bacillus cereus* (Table 1). Although TM2 and SPS3 were most closely aligned to *B. cereus* with 99% similarity, 25 nucleotides of 16S rDNA between TM2 and SPS3 were different from each other. After 20 h of incubation on nutrient agar, colonies of TM2 and SPS3 formed on the agar were examined, and their characteristics were tabulated in Table 2. The examinations of cell morphology, Gram reaction and spore formation were also conducted, and TM2 and SPS3 revealed their unique characteristics, as shown in Table 2.

**Table 1:** **Identification of microorganisms isolated from the tidal mud and shrimp pond bottom soil**

Isolate	Length ^*^(bp)	GenBank accession no.	Identification	Similarity (%)
TM2	1474	JX544748.1	*Bacillus cereus*	99
SPS3	1470	JX993816.1	*Bacillus cereus*	99

^*^ Fragment length of PCR product (16S rDNA).

**Table 2:** **Characteristics of isolated strains**

Characteristic	Isolate	
	TM2	SPS3
Cell size (μm)	Width: 1.0 ~ 1.2, Length: 4.0 ~ 5.0	Width: 1.0 ~ 1.2, Length: 3.0 ~ 4.0
Cell shape	Rod, chain (4 ~ 6 cells)	Rod, V-shaped pairs
Motility	Motile	Motile
Gram reaction	+	+
Spore formation	+ (ellipsoidal, central or paracentral)	+ (ellipsoidal, central or paracentral)
Colony size (cm)	0.5 ~ 0.65	0.65 ~ 0.8
Colony color	Light cream	Off-white
Colony shape	Circular	Irregular
Colony edge	Undulate	Undulate
Colony texture	Streaky	Non-streaky

### Measurement of reducing sugar

**Figure 1:**
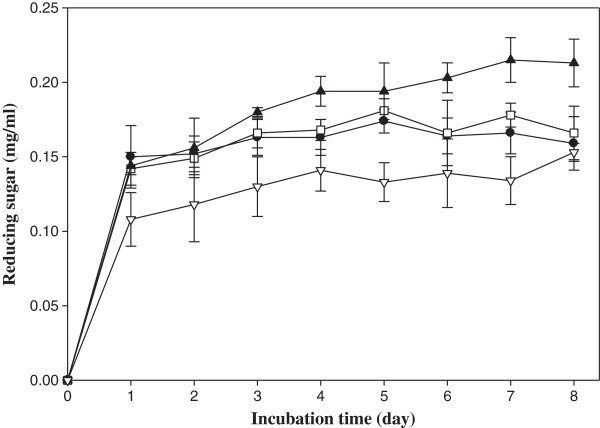
**Time courses of reducing sugar production by the strains TM2 (●)** ***,*** **SPS3 (□), EW5 (▲) and KA1 (▽) in 1% SSP medium incubated at 37°C (TM2 and SPS3) and 47°C (EW5 and KA1) in a shaking incubator at 170 rpm.** The data are presented as the mean ± SD (n = 3).

All seven strains were tested for their ability to degrade SSW in a 10-ml tube. The degradation of SSP was revealed by the production of reducing sugar. All strains produced reducing sugar in 5 ml of SSP broth (data not shown). TM2 and SPS3, among the new isolates, and EW5 and KA1, from the lab-stored strains, were found to be potential candidates that could degrade SSW, based on the amount of reducing sugar produced. During 8 days of incubation, the greatest quantity of reducing sugar (0.22 mg/ml) was produced by the strain EW5 after 7 days of incubation followed by SPS3 (0.18 mg/ml) and TM2 (0.17 mg/ml) after 5 days and KA1 (0.15 mg/ml) after 8 days of incubation (Figure 1). This might be due to the endolytic nature of its enzyme, which most likely degraded SSP to oligosaccharides rather than monosaccharides (Brzezinska et al. 2013; Wang et al. 2012). In contrast, EW5 produced no clear zone but did produce the highest amount of reducing sugar, indicating the production of an exolytic enzyme by this strain that most likely degraded SSP to mono- and disaccharides.

### Kinetics of the SSW biodegradation

**Figure 2:**
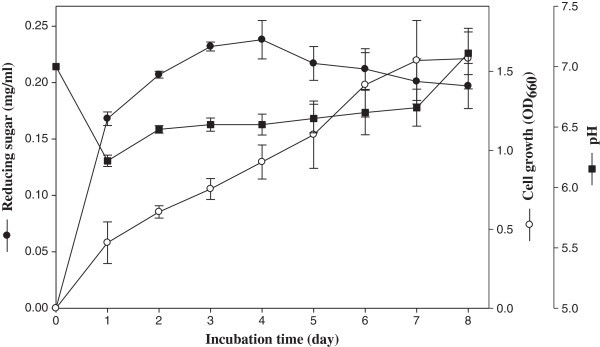
**Time courses of shrimp-shell waste biodegradation by** ***B. cereus*** **EW5 in a 250-ml flask incubated at 47°C.** The data are presented as the mean ± SD (n = 3).

After checking the SSW degradation ability of the four strains, EW5 was identified as the most promising candidate. Therefore, this strain was selected for further biodegradation analysis in 100 ml of SSP medium. The kinetics of biodegradation was studied in terms of conversion efficiency of SSW to reducing sugar, cell density and pH level during 8 days of incubation (Figure 2). During biodegradation, the exponential growth of strain EW5 was observed up to 7 days and after then stationary phase was achieved. At the same time, a rapid pH drop from 7.0 to 6.22 was observed during the first day of incubation, and the pH then gradually increased up to 7.11. The rapid fall of pH during the first day might be due to the ability of strain EW5 to use chitin as a substrate for its growth and simultaneously to produce acids via pyruvate (Bellaaj et al. 2012a). With the course of fermentation, the pH of the medium increased possibly due to accumulation of chitosaccharides, which contain an amino group (Halder et al. 2013). A slight decrease of pH during the first day and then an increasing pattern was also reported by Wang et al. ( 2009b) in a culture of *B. cereus* TKU018 on SSP media. The saccharification of SSP increased with the incubation period up to 4 days (0.24 mg/ml) and then slowly decreased up to 8 days. Strain EW5 most likely produced the highest amount of extracellular chitinolytic enzyme in the middle of the exponential growth phase, as indicated by the higher amount of reducing sugar production during days 3–5 of incubation. Approximately 24 mg of reducing sugar could be recovered per gram of dried SSW, indicating a 2.4% recovery. Halder et al. ( 2013) reported the production of 5.5 mg of chitosaccharides per gram of SSW fermented by *A. hydrophila*. The decrease of reducing sugar from the sixth day of incubation might be due to the utilization of some sugar by the strain for growth following the depletion of the initial substrate. Wang et al. ( 2009b) also reported increased reducing sugar content during the initial 3 days of fermentation of squid pen powder by *B. cereus* and a slight lowering thereafter. In our previous study, we demonstrated that *B. cereus* produces a proteolytic enzyme (Kim et al. 2010). Therefore, in this study, the decrease in the amount of reducing sugar at the end of exponential growth phase could also be due to decreased activity of chitinolytic enzyme hydrolyzed by the protease activity of the same strain. Liang et al. ( 2012) also reported that *B. cereus* is a protease- and chitinase-producing strain, and they demonstrated the decreased chitosanase activity with the appearance of its protease activity while degrading shrimp head waste. The action of protease on chitinase making it unavailable for further action on the substrate was also reported by Nawani et al. ( 2010).

### Production of chitosaccharides

**Figure 3:**
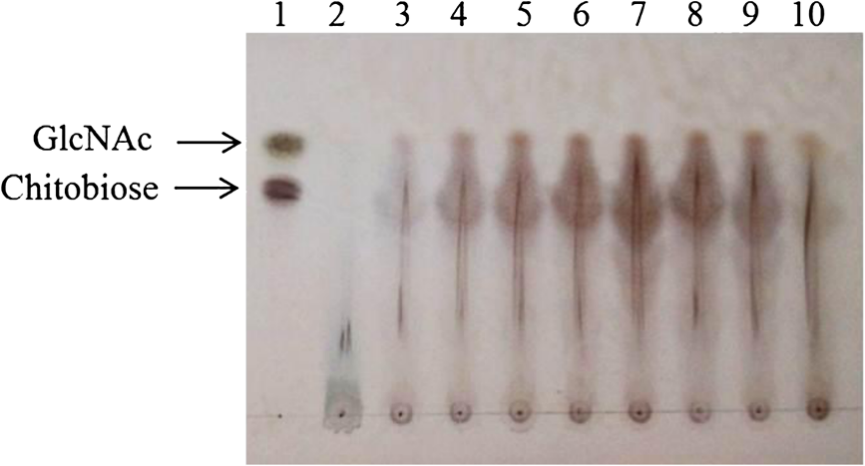
**TLC analysis of shrimp-shell waste degradation.** Lane 1: mixture of GlcNAc and chitobiose (0.2%), Lane 2: Day 0 culture supernatant, Lanes 3–10: days 1–8 culture supernatant of strain *B. cereus* EW5 in 1% SSP medium.

In our study, the degradation of SSW by EW5 exhibited simultaneous production of GlcNAc and chitobiose that was revealed by TLC (Figure 3). Clear bands of GlcNAc and chitobiose appeared on the TLC plate in lanes 4–9, which matched the results of the reducing sugar production assays presented in Figure 2, and similar results was obtained in a study by Halder et al. ( 2013). The production of chitin oligosaccharides, including chitobiose, by *B. cereus* fermentation of shrimp-head waste has also been reported by researchers. Liang et al. ( 2012) found 0.20 mg/ml of chitobiose in culture supernatants, but there was no production of GlcNAc. Wang et al. ( 2012) also reported that there was no production of GlcNAc during *B. cereus* TKU027 fermentation. On the other hand, Suresh ( 2012) reported the production of GlcNAc for the first time via solid-state fermentation of shrimp waste using five strains of bacteria separately. He recorded a maximum 4.7 μmol of GlcNAc/g of dry substrate produced by *Vibrio* species after 4 days of incubation. However, to the best of our knowledge, until now, there have been no reported data about the fermentation production of GlcNAc from SSW by *B. cereus.*

### Antioxidant activity of biodegraded SSW

Currently, there is a strong need for effective antioxidants from natural sources as alternatives to synthetic antioxidants to prevent free radical-induced diseases such as cancer, cardiovascular disease, age-related macular degeneration and other such diseases (Ramakrishna et al. 2012). It is well established that antioxidants can scavenge the free radical chain of oxidation and form stable free radicals, which prevents further oxidation. To increase the reutilization value of SSW, it was degraded by the strain EW5 for 8 days, and the antioxidant activity of the culture supernatant was subjected to a DPPH free radical scavenging assay, an ABTS radical cation decolorization assay, a hydroxyl radical scavenging assay and a reducing power assay to evaluate its different antioxidant properties.

### DPPH (2, 2-diphenyl-1-picrylhydrazyl) free radical scavenging activity

**Figure 4:**
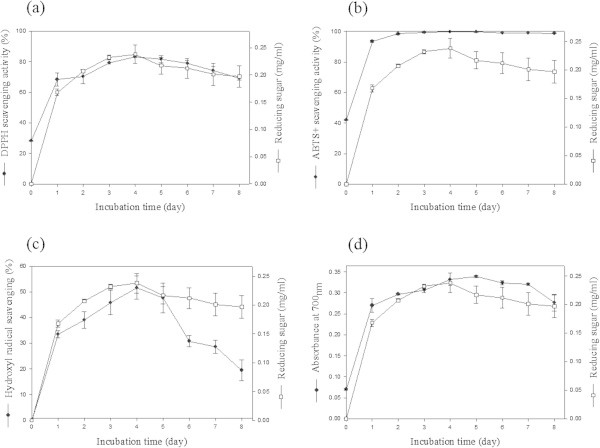
**Antioxidant activity of the culture supernatant collected during the biodegradation of shrimp-shell waste by the strain** ***B. cereus*** **EW5. (a)** DPPH free radical scavenging activity, **(b)** ABTS radical scavenging activity, **(c)** hydroxyl radical scavenging activity, and **(d)** reducing power. The data are presented as the mean ± SD (n = 3).

The DPPH radical is used to measure the free-radical scavenging capacity of antioxidants extensively and has been reported as being more specific for lipophilic antioxidants (Prior et al. 2005). The lower absorbance of the reaction mixture indicates higher free radical scavenging activity. As shown in Figure 4a, the DPPH free radical scavenging ability of the culture supernatant was between 68.5 and 83.4% during the 8 days of incubation. The day 4 culture supernatant displayed both the highest reducing sugar and highest scavenging ability for DPPH, which was comparable to the scavenging ability of 0.1 mM L-Ascorbic acid (82.27%). The DPPH radical scavenging ability was also demonstrated to be 70-75% and 56% in culture supernatants from squid pen powder (Wang et al. 2009b) and SSP (Wang et al. 2009a), respectively, fermented by *B. cereus* species. Annamalai et al. ( 2011) also reported a 75% DPPH radical scavenging activity for an *Alcaligenes faecalis* AU02 culture supernatant grown on SSP. Therefore, our culture broth displayed a higher level of DPPH scavenging activity compared with the results of these studies.

### ABTS radical cation decolorization assay

The ABTS radical cation scavenging activity of the culture supernatant was recorded to be between 93.42 and 99.62% during 8 days of incubation (Figure 4b). L-Ascorbic acid (0.3 mM), which was used as a positive control, displayed 71.39% ABTS radical scavenging activity. ABTS has been reported for both lipophilic and hydrophilic antioxidants (Prior et al. 2005). In our study, the ABTS radical scavenging activity of the culture supernatant was stronger than the DPPH radical scavenging activity, with 99.6% scavenging measured after 4 days of incubation. Similar findings were also reported by Sachindra and Bhaskar ( 2008). They reported 94.81% ABTS radical scavenging activity for the lyophilized powder of liquor from fermented shrimp waste, which was higher than the DPPH radical scavenging activity.

### Hydroxyl radical scavenging activity

The hydroxyl radical is a highly reactive oxidizing species that can react with most biomolecules and is responsible for the formation of other radicals (Sachindra and Bhaskar, 2008). In our study, the hydroxyl radical scavenging activity of the culture supernatant was between 19.43 to 51.56% during the 8 days of incubation (Figure 4c). The maximum scavenging activity was observed in the culture supernatant incubated for 4 days. L-Ascorbic acid (0.1 mM), which was used as a positive control, displayed 45.52% scavenging activity. The hydroxyl radical scavenging activity in our study was comparable with that of Nawani et al. ( 2010) who demonstrated 57% hydroxyl radical scavenging activity of chitobiose purified from shrimp- and crab-shell waste by chitinases and the proteases of *Microbispora* species and *Bacillus* species. They also demonstrated a linear increase in the antioxidant activity with the increase in chitinase from the *Bacillus* sp.

### Reducing power assay

The reducing power assay is used to evaluate the ability of an antioxidative compound to donate electrons or hydrogen and serves as a significant indicator of potential antioxidant activity (Gao et al. 2012). Several studies have reported that the antioxidative effect is related to the development of reductones (Yen and Duh 1993) and directly correlated with the reducing power of certain bioactive compounds (Bellaaj et al. 2012a). During the reducing power assay, the presence of reductants in the culture supernatant causes the reduction of ferric cyanide complex to a ferrous form. During the 8 days of incubation, the reducing power of the culture supernatant was between 0.23 and 0.34 at A_700nm_ (Figure 4d). The absorbance of the control at 700 nm was recorded as 0.03. The increase in the absorbance indicates that reducing power increased. The maximum reducing power of the culture supernatant was reached after 5 days of incubation and was recorded to be 0.34 (A_700nm_) at a 0.22 mg/ml reducing sugar concentration, which was higher than the reducing power of shrimp waste hydrolysate by *B. cereus* at a 0.25 mg/ml concentration reported by Bellaaj et al. ( 2012b).

In this study, the SSP culture medium was pretreated with NaOH and HCl for deproteinization and demineralization, respectively. Wang et al. ( 2011) reported that SSW is a rich source of phenolic compounds that play an important role in antioxidant properties. Therefore, the antioxidant activity of the culture medium (day 0) was also analyzed. The results indicated that the DPPH and ABTS radical scavenging activity of the untreated medium were 6.98% and 4.14%, respectively, and for the pretreated medium were 28.29% and 41.87%, respectively. The pretreated medium also displayed very little reducing power (0.07) and no hydroxyl radical scavenging ability. The above data indicate that a very small level of antioxidant activity of SSP medium was increased by autoclaving, which was further increased to a larger extent by the pretreatment. These antioxidant activities might result from the greater or lesser exposure of chitinous materials due to autoclaving and the degradation of chitin to some extent during the pretreatment of SSP. Wang et al. ( 2009b) also reported 15-20% DPPH radical scavenging activity of autoclaved medium containing SSP. It has been reported that chitin, chitosan and peptide have antioxidative properties (He et al. 2006). However, as shown in Figure 4, most of the antioxidant activity was achieved after fermentation of SSP by the strain *B. cereus* EW5.

In our study, it was observed that the antioxidant activity of the culture supernatant increased with increasing amounts of reducing sugar. Wang et al. ( 2009b) also demonstrated a positive correlation between antioxidant activity and reducing sugar content in a culture of *B. cereus* on squid pen-containing media. Some other researchers have also reported the concentration-dependent manner of radical scavenging activities (Bellaaj et al. 2012b; Sachindra and Bhaskar 2008). Many researchers have reported that the antioxidant activity might be due to the bioactive compounds, including phenolics, chitooligosaccharides, oligopeptides, peptides and free amino acids, present in the culture supernatant that are most likely produced during the fermentation of shrimp waste (Bellaaj et al. 2012a; Sachindra and Bhaskar 2008; Wang et al. 2009a). However, in this study, the major contributor to the antioxidant activity was not chitooligosaccharides or peptides. The compounds produced in the culture supernatant were identified by TLC as GlcNAc and chitobiose, which are most likely the main contributors to the antioxidant activity of the culture supernatant. Nawani et al. ( 2010) also demonstrated the antioxidant activity of chitobiose purified from shrimp- and crab-shell waste. The above findings suggest that *B. cereus* EW5 culture supernatant has a strong ability to donate electrons to reactive free radicals, converting them into more stable products and terminating the free radical chain reaction. Therefore, the supernatant contains good natural antioxidant candidates.

### Protective effect against DNA damage

**Figure 5:**
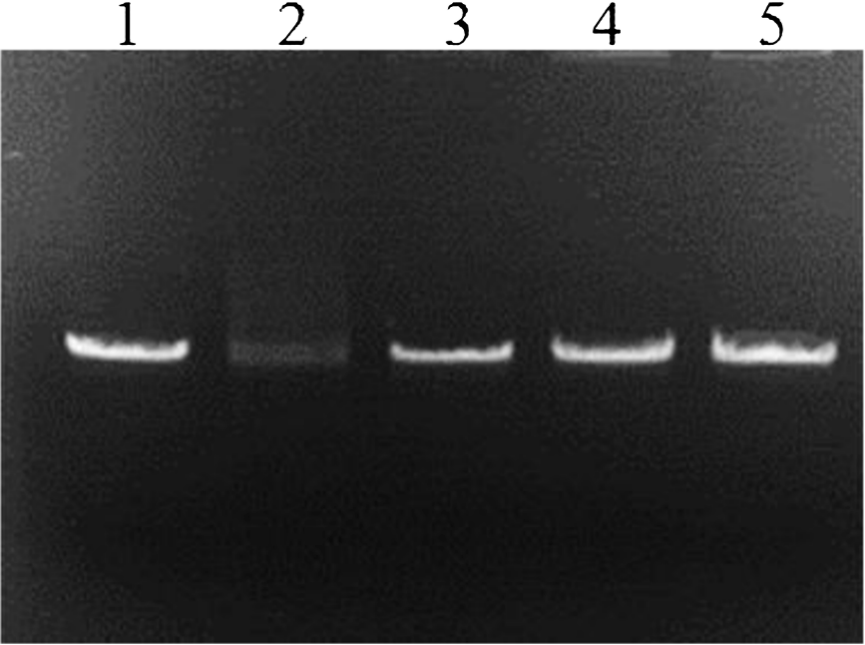
**Electrophoresis of λ DNA demonstrating protective effect of strain EW5 culture supernatant.** Lane 1: undamaged DNA, Lane 2: DNA exposed to Cu (II) and ascorbic acid, Lane 3–5: DNA exposed to Cu (II) and ascorbic acid in the presence of 50, 100 and 150 μl of culture supernatant.

Currently, there is a great interest in evaluating the protective activity of natural antioxidant compounds against damaging effects on important cellular components. Free radical-induced damage to DNA has been explained by the reaction of hydroxyl radicals with guanine, leading to mutation (Saenjum et al. 2010) or the death of cells (Kim et al. 2012). In this study, to assess the DNA protective effect of EW5 culture supernatant, hydroxyl radical-induced DNA damage was conducted using copper (II) sulfate and ascorbic acid. The reaction was conducted both in the presence and absence of culture supernatant. The λ DNA treated with hydroxyl radical in the presence of culture supernatant displayed a clear band, whereas in the absence of culture supernatant, the DNA produced only a smear (Figure 5). These results suggest that the EW5 culture supernatant has a strong protective effect on DNA damage from hydroxyl radicals. In this assay, a significant protective action of the culture supernatant with a dose of 100 μl was clearly visible on 1% agarose gel. The DNA was also partially protected even with a dose of 50 μl, whereas it was almost completely damaged in the absence of culture supernatant (Figure 5). The DNA protective activity of astaxanthin extracted from shrimp-shell waste against degradation by hydroxyl radicals was reported very recently by Sila et al. ( 2013). However, to the best of our knowledge, there is no report on the DNA protective activity of SSW degraded by *B. cereus.*

## Conclusion

In conclusion, a potential chitin-degrading strain, *B. cereus* EW5 demonstrated the highest SSW degradation ability, and the SSW degradation resulted in the production of GlcNAc and chitobiose, which exhibited strong antioxidant activity and DNA protection ability. These results suggest the broad potential for the environmentally friendly application of this strain to the recovery of natural antioxidants from SSW, which will not only add its reutilization value but also solve the environment pollution problem caused by shrimp processing waste. To our knowledge, this is the first scientific report about the production of GlcNAc and inhibition of DNA damage from SSW by *B. cereus*. Further study is warranted to improve productivity and optimize a scaled-up process.

## Materials and methods

### Preparation of the shrimp-shell powder (SSP)

Frozen Pacific white shrimp (*Litopenaeus vannamei*) was purchased from a local market. The SSW (carapace, body shell and tail hulls) was washed thoroughly with tap water, boiled for 15 minutes and dried in an oven for 12 h at 120 ± 1°C. The dried shells were ground and sieved to powder with a particle size of less than 63 μm and stored at 4°C until use.

### Pretreatment of the SSP

Before preparation of the culture medium, the SSP was pretreated in an aqueous solution with NaOH at pH 12.6 ± 0.2 on a hot plate maintained at 80 ± 5°C with slow stirring for 5 h for deproteinization. After treatment with NaOH, the SSP was treated with HCl at pH 4.0 ± 0.2 at room temperature and was continually stirred overnight for demineralization.

### Isolation and screening of chitin-degrading strains

Tidal mud and pond bottom soil samples were collected from Nakdong River estuary (Busan, Korea) and Pilgyeong-Susan shrimp farm (Namhae, Korea). Approximately one gram of soil sample was inoculated into 100 ml of nutrient broth and incubated at 37°C and 170 rpm in a conical flask (500 ml) for 20 h. Then, 5 ml of the culture broth was inoculated into 50 ml of SSP medium (0.8% SSP, 0.5% NH_4_Cl, 0.1% K_2_HPO_4_ and 0.05% MgSO_4_.7H_2_O, pH 7.0) in a conical flask (250 ml) and incubated at 37°C and 170 rpm. The microbial population was observed under a microscope once daily. After 8 days of incubation, 10^-4^ and 10^-5^ dilutions of SSP broth were poured on nutrient agar plates and incubated at 37°C. After 20 h of incubation, all types of colonies were sub-cultured separately in glass tubes containing 3 ml of nutrient broth. The strains obtained from this screening were sub-cultured repeatedly in nutrient agar plate to obtain pure cultures.

The isolated strains were screened on agar plates containing 0.8% SSP as the sole carbon source (0.5% NH_4_Cl, 0.1% K_2_HPO_4_, 0.05% MgSO_4_.7H_2_O and 1.25% agar powder, pH 7.0). The plates were incubated at 37°C for 2 days. To extend the screening possibility, eight protein- and/or lipid-degrading strains stored in our laboratory were also tested for their chitin-degrading ability on SSP agar.

### Chitinase activity test

The chitin-degrading ability of isolates taken from the tidal mud and shrimp-pond bottom soil was investigated by plating bacteria on SSP agar containing 0.8% SSP and incubating for 4 days at 37°C. Eight lab-stored strains were also investigated at 47°C in parallel. Chitin hydrolysis was assayed after overlaying Petri dishes with 10 ml of Lugol’s solution for 5 min. A positive reaction was indicated by a clear zone (light orange color) around the bacterial colony. The diameter of each clear zone was measured for a qualitative evaluation of chitinase activity.

### Measurement of reducing sugar

Isolates carrying chitinase activity were cultured separately in 10-ml tubes with 5 ml of SSP broth containing 1% SSP. After incubation for 1 to 8 days, the culture broths were centrifuged at 10,000 rpm and 4°C for 10 min, and the supernatants were collected for the colorimetric measurement of reducing sugar by the modified method of Imoto and Yagishita ( 1971) with GlcNAc (Sigma-Aldrich Co., St. Louis, MO, USA) as a reference compound. Briefly, 1 ml of the color reagent was mixed with 200 μl of culture supernatant. The mixture was incubated in boiling water in an Eppendorf tube for 8 min. After cooling at room temperature, the absorbance of the mixture at 405 nm (*A*_405_) was read in a 96-well microplate using ELISA (BioTek EL800, USA). The decrease in *A*_405_ was employed to determine the reducing sugar using a standard curve.

### Identification of isolated useful strains

After screening of isolates by the clear zone assay and reducing sugar measurements, potential chitin-degrading strains were primarily characterized by colony and cell morphology under microscopy, motility and Gram staining. For final identification, 16S rDNA sequence analysis was conducted. Genomic DNA was extracted with an AccuPrep® Genomic DNA extraction kit (Bioneer, Korea), according to the manufacturer’s instructions. PCR amplification of the DNA using the universal 16S rDNA primer sets, 27 F (5’-AGAGTTTGA TCMTGGCTCAG-3’) and 1492R (5’-TACGGYTACCTTGTTACGACTT-3’), was performed with a PCR thermal cycler DICE model TP600 (TaKaRa, Japan). PCR was performed as follows: initial denaturation at 94°C for 5 min, 30 cycles of denaturation at 94°C for 30 s, annealing at 55°C for 30 s, extension at 72°C for 30 s and a final extension at 72°C for 5 min. The sequencing of the PCR products was performed by Macrogen Ltd. (Seoul, Korea). The sequences of the 16S rDNA were compared with the available sequences in the NCBI GenBank using the Advanced Basic Local Alignment Search Tool (BLAST) similarity search option accessible from the homepage at the National Center for Biotechnology Information (http://www.ncbi.nlm.nih.gov/). The ClustalW program of BioEdit Sequence Alignment Editor Version 7.0.9 was used to check alignment. After identification, the isolated strains were stored in 25% glycerol at -70°C for further use.

### Biodegradation of SSW

The strain exhibiting the highest chitin-degrading ability was cultured in a 250-ml conical flask containing 100 ml of SSP medium (1% SSP, 0.5% NH_4_Cl, 0.1% K_2_HPO_4_ and 0.05% MgSO_4_.7H_2_O, pH 7.0). The SSP medium was incubated at 47 ± 1°C and 170 ± 5 rpm up to 8 days. After every 24 h, the culture supernatant was collected (10,000 rpm and 4°C for 10 min) from the flask for analysis of biodegradation. The experiment was conducted in triplicate.

### Product analysis by thin layer chromatography

Degradation of SSW from the selected strain was analyzed using thin layer chromatography (TLC). The culture supernatant collected from the flask was concentrated to 1/5 of the original volume and applied 10 times (1 μl each) onto TLC Silica Gel 60 plate (Sigma–Aldrich, Germany) and then chromatographed two times (1 h each) in a mobile phase containing 5:4:2:1 (v/v/v/v) ratio of n-butanol:methanol:28% aqueous ammonia solution:water (Songsiriritthigul et al. 2010). The products were stained using a mixture of acetone (4 ml), diphenylamine (80 mg), aniline (80 μl), and 85% orthophosphoric acid (600 μl) (Brunel et al. 2013) followed by baking at 115 ± 2°C for 15 min. A mixture of GlcNAc and N, N’-Diacetylchitobiose (Chitobiose) solution (0.2%) was also run alongside as a marker.

### Determination of antioxidant activity

#### DPPH (2, 2-diphenyl-1-picrylhydrazyl) radical scavenging assay

The DPPH free radical scavenging ability of the culture supernatant was determined as described by Blois ( 1958) with some modification. Two milliliters of 0.1 mM DPPH solution in 80% ethanol was added to 1 ml of the culture supernatant. The mixture was kept at room temperature (25°C) in the dark for 30 min, and the absorbance was measured at 517 nm (Opron-3000® UV/VIS Spectrophotometer, Hanson technology Co., Ltd., Korea) against a blank sample. The sample blank was prepared by replacing DPPH with 80% ethanol. The DPPH radical scavenging activity was determined using the following formula:


A control sample was prepared by mixing 1 ml of 80% ethanol with 2 ml of 0.1 mM DPPH. L-Ascorbic acid (0.1 mM) was used as a positive control under the same assay conditions. The experiment was conducted in triplicate.

#### ABTS radical cation decolorization assay

The ABTS radical cation decolorization assay was conducted according to the method of Re et al. ( 1999) with some modifications. The ABTS radical cation (ABTS reagent) was prepared by mixing 5 ml of 7 mM ABTS (2, 2’-Azino-bis3-ethylbenzothiazoline-6-sulfonic acid) with 5 ml of 4.9 mM Potassium persulfate (K_2_S_2_O_8_). The mixture was kept in the dark at room temperature for 16 h. The absorbance of ABTS reagent was then adjusted to 0.720 ± 0.02 at 734 nm with 80% ethanol. To determine the scavenging activity, 900 μl of ABTS reagent was added to 100 μl of culture supernatant, and the absorbance was measured at 734 nm after a 6 min interval. L-Ascorbic acid (0.3 mM) was used as a positive control. The percentage inhibition of the sample was calculated using the following equation:


The control sample was prepared by replacing the culture supernatant with distilled water (DW). The sample blank was prepared by replacing the ABTS reagent with 80% ethanol. The analysis was conducted in triplicate.

#### Hydroxyl radical scavenging activity

Hydroxyl radical scavenging activity was determined according to Beara et al. ( 2009) with some modification. A half milliliter of the culture supernatant was mixed with 250 μl of ortho-phenanthroline (7.5 mM), 1.25 ml of phosphate buffer (0.2 M, pH 6.6), 250 μl of ferrous sulfate (7.5 mM) and 250 μl of H_2_O_2_ (0.5%) and diluted to a final volume of 6.25 ml with DW. The solution was mixed vigorously and incubated at room temperature for 30 min. After incubation, the absorbance was measured in a 96-well microplate at 490 nm using an ELISA. The scavenging percentage (P%) was calculated using the following formula:


where A is the absorbance value of all solutions, including H_2_O_2_ and the sample, A1 is the absorbance value without the sample and A2 is the absorbance value without H_2_O_2_ and the sample.

#### Reducing power assay

Reducing power was determined by the method prescribed by Wu et al. ( 2010) with some modification. One milliliter of culture supernatant was mixed with 1.0 ml of 0.2 M phosphate buffer (pH 6.6) and 1.0 ml of 1% potassium ferricyanide. The reaction mixture was incubated at 50°C for 20 min in a shaking incubator. After incubation, the reaction was stopped by adding 1.0 ml of 10% (w/v) trichloroacetic acid to the reaction mixture and centrifuged at 3,000 rpm for 10 min. From the upper layer, 2 ml of solution was taken and mixed with 2 ml of DW and 0.4 ml of 0.1% FeCl_3_. The mixture was incubated for 10 min at room temperature. After 10 min, the absorbance of all sample solutions was measured at 700 nm. An increase of absorbance indicated an increase in reducing power. The control sample was prepared by replacing the culture supernatant with DW. The test was performed in triplicate.

### Determination of DNA protective activity

The DNA protective activity of the EW5 culture supernatant was examined according to the method described by Kim et al. ( 2012). The λ DNA (4 μg) was exposed to the action of hydroxyl radicals generated by the mixture of L-Ascorbic acid (1 mM final concentration) and copper (II) sulfate (0.1 mM final concentration) in the presence and absence of EW5 culture supernatant. Three different amounts, such as 50, 100 and 150 μl, of day 4 culture supernatant were used to evaluate their DNA protective activity. DW was used as a control. The mixture was incubated at 37°C for 1 h. An aliquot of 10 μl was loaded onto a 1% agarose gel in 1 × TAE buffer, and electrophoresis was conducted at 100 V for 25 min. The DNA bands were visualized using ethidium bromide under a UV transilluminator and documented using a Polaroid webcam.
